# Chronic-Care-Management Programs for Multimorbid Patients with Diabetes in Europe: A Scoping Review with the Aim to Identify the Best Practice

**DOI:** 10.1155/2021/6657718

**Published:** 2021-11-09

**Authors:** Julia Heike Brettel, Ulf Manuwald, Henriette Hornstein, Joachim Kugler, Ulrike Rothe

**Affiliations:** Technische Universität Dresden, Faculty of Medicine “Carl Gustav Carus”, IPAS/Health Sciences/Public Health, Fetscherstraße 74, 01307 Dresden, Germany

## Abstract

**Aim:**

This scoping review is aimed at providing a current descriptive overview of care programs based on the chronic care model (CCM) according to E. H. Wagner. The evaluation is carried out within Europe and assesses the methodology and comparability of the studies.

**Methods:**

A systematic search in the databases PubMed, Embase, and MEDLINE via OVID was conducted. In the beginning, 2309 articles were found and 48 full texts were examined, 19 of which were incorporated. Included were CCM-based programs from Belgium, Cyprus, Germany, Italy, Switzerland, and the Netherlands. All 19 articles were presented descriptively whereof 11 articles were finally evaluated in a checklist by Rothe et al. (2020). In this paper, the studies were tabulated and evaluated conforming to the same criteria.

**Results:**

Due to the complexity of the CCM and the heterogeneity of the studies in terms of setting and implementation, a direct comparison proved difficult. Nevertheless, the review shows that CCM was successfully implemented in various care situations and also can be useful in single practices, which often dominate the primary care sector in many European health systems. The present review was able to provide a comprehensive overview of the current care situation of chronically ill patients with multimorbidities.

**Conclusions:**

A unified nomenclature concerning the distinction between disease management programs and CCM-based programs should be aimed for. Similarly, homogeneous quality standards and a Europe-wide evaluation strategy would be necessary to identify best practice models and to provide better care for the steadily growing number of chronically multimorbid patients.

## 1. Introduction

Demographic change and the increasing number of patients with multiple chronic conditions will face a major challenge for the current health care system in Germany shortly. The current models, which mostly deal with individual diseases in particular, often use so-called disease management programs (DMPs). Recent publications showed that the need for programs that cover patients suffering from several chronic diseases is continuously growing. One possible model for the managed care of multimorbid patients with several chronic diseases is the widely accepted chronic care model (CCM) by Wagner et al. [1–3].

**Figure 1:**
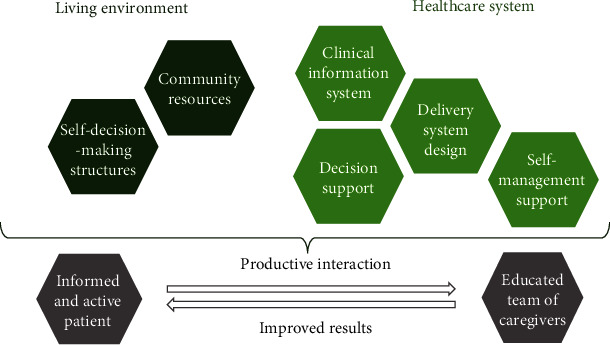
Graphic illustration of the CCM (according to [5]).

The CCM integrates 6 key elements that are designed to optimize the coordination of care and the treatment, information, and motivation of multimorbid patients. The fundamental aspects of the CCM described in Figure 1 are health care organization, delivery system design, clinical information systems, decision-support, self-management support, and community resources [1–4]. Therefore, the objective of this scoping review was to provide an actual descriptive overview of published chronic care management program evaluations accomplished within Europe using the CCM and to identify the best practice model.

## 2. Materials and Methods

### 2.1. Databases and Selection Criteria

The search for suitable publications was carried out in May 2018. The databases PubMed, Embase, and MEDLINE via OVID were used for research. In addition to the electronic database search, searches were conducted in bibliographies of relevant publications and on the websites of the institutions. We included all European studies that were published in English, with multimorbid participants older than 15 years. In this case, multimorbidity means at least two chronic diseases, for example, diabetes mellitus and CVD. The search was limited to studies published between January 2008 and May 2018 (Table 1).

### 2.2. Search Strategy

The search string was designed to be as open as possible, since not every included study contains all elements of CCM, and their names may vary from study to study. Different word combinations were used and combined with the operators AND and OR. Inclusion and exclusion criteria were also defined (Table 1). To improve the quality of the results, British and American English terms were used.

The following search string for PubMed was used: ((“chronic”[Text Word] OR “morbid”[Text Word] OR “comorbid”[Text Word] OR “multimorbid”[Text Word]) AND (“chronic”[Text Word] OR “comorbid”[Text Word] OR “multimorbid”[Text Word] OR “morbidity”[Text Word] OR “comorbidity”[Text Word]) AND (“care management”[Text Word] OR “care model”[Text Word] OR “care program”[Text Word] OR “care programme”[Text Word] OR (“care”[All Fields] AND “programmes”[Text Word]) OR “care programming”[Text Word] OR “care programs”[Text Word] OR “chronic care management”[Text Word] OR “chronic care model”[Text Word] OR “chronic care program”[Text Word] OR “chronic care programme”[Text Word] OR “chronic care programmes”[Text Word] OR “chronic care programs”[Text Word] OR “chronic care”[Text Word] OR (“chronic”[Text Word] AND “care”[Text Word] AND “management”[Text Word]) OR (“chronic”[Text Word] AND “care”[Text Word] AND “model”[Text Word])) AND 2008/01/01 : 2018/05/01[Date - Publication] AND “English”[Language] AND (“aged”[MeSH Terms] OR “adult”[MeSH Terms] OR “adult”[MeSH Terms]) AND “Europe”[MeSH Terms]) NOT “systematic”[Filter].

In contrast to the search in PubMed, the search for results in Embase and MEDLINE via Ovid was modified and carried out without MeSH terms: (Chronic or Comorbid or Morbid or Multimorbid).tw; (Care management or Care program or Care model).tw; (chronic care management or chronic care model or chronic care program).tw; 2 or 3; 1 and 4; chronic care model.tw; 5 and 6; limit 7 to english language; limit 8 to humans; limit 9 to yr=“2008 - Current”; remove duplicates from 10.

### 2.3. Selection Process and Data Extraction

The PSI scheme, which is suitable for scoping reviews, was used to identify the appropriate papers, and the commonly used PICO has been replaced as it is intended for systematic reviews. To optimize the quality of the studies, we expanded the PSI scheme. The items quality and outcome were added.

The form of diabetes mellitus type 2 (T2DM) is a special case since 80% of cases of this disease are associated with comorbidities and therefore cannot be clearly distinguished from the literature [6, 7]. Consequently, the chronic disease diabetes mellitus was included in the modified PSI scheme: P: patients—people with more than one disease, diabetes mellitus, exist between the ages of 15 and 99 years; S: study design—study design unlimited; I: interventions—implementation and evaluation of the chronic care model; O: outcome—improvement of the state of health and clinical outcomes; Q: quality—sufficient quality and traceability in operationalization, method, and presentation of results.

We also focused on the methodological quality of the assessment and on the comparability of the included studies. All studies and programs have been evaluated using a checklist provided by Rothe et al. published in 2020 [8]. Subsequently, the extraction of the data was performed by four independent reviewers.

## 3. Results

### 3.1. Overview of Included Studies

**Figure 2:**
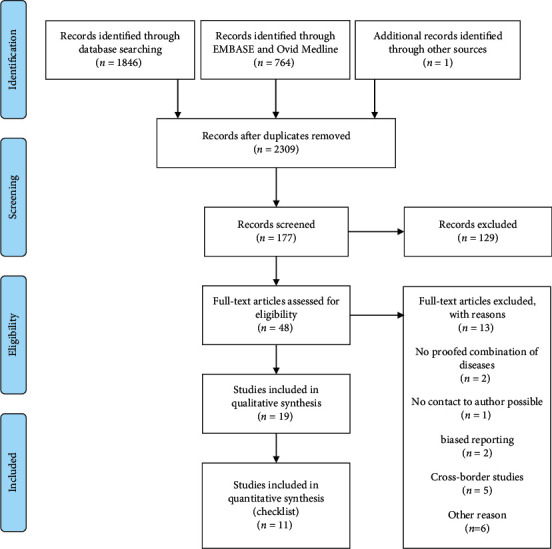
PRISMA flow diagram for searching strategy [28].

The PRISMA flow chart, shown in Figure 2, describes the screening process. In the beginning, we found 2309 publications, detected 19 suitable publications, and finished the extraction of the data within the checklist with 11 articles [9–19]. Out of 2309 titles and abstracts, 48 full texts were screened for inclusion. Ultimately, 19 publications met all inclusion criteria [9–27]. Programs from Belgium, Cyprus, Germany, Italy, the Netherlands, and Switzerland were found using the CCM. The results are discussed descriptively in Results and tabulated additionally. Due to the complexity of the CCM, the heterogeneity of the studies about the study environment and different study designs that led to bias, a comparison, and a uniform assessment turned out to be difficult. Therefore, it is necessary to consider each particular study separately and to analyze it in the overall context of the respective setting (country, location, supply situation, etc.). A uniform strategy for the conduct of studies would be desirable to better assess and optimize the implementation of the CCM in the future. The included items are shown in Table 1. It was possible to sort the publications either by study quality or by country. Of the 19 studies, only two studies were designed as randomized control trials. Four studies were longitudinal and cohort studies, and 4 studies were cross-sectional studies. Besides, one controlled study and one quasiexperiment were published in the same period. Also, two written and one oral interview, as well as 4 study protocols, were carried out but not included in the evaluation due to the poor quality of the studies.  Table 2 provides a detailed overview of the countries in which the studies were published and their study design.

### 3.2. Synthesis of the Results

To determine the “best practice,” 11 of the studies were specifically examined [9–19]. For this purpose, the implementation of the pillars of the CCM was assessed on its own elements and then those of the checklist according to Rothe et al. [8]. The detailed evaluation of the studies can be found in Table S1 in the supplementary material. The selected studies are compared with each other using the checklist, which provides an overview of the field of study, including its strengths and limitations. A sole concentration on the elements of the CCM would have been too unspecific due to the complexity of the model and its multifaceted implementation in practice. Furthermore, the following essential questions regarding the implementation of the CCM in Europe [29] were answered.

#### 3.2.1. Are There Currently Studies on CCM-Based Programs in Europe and Have They Been Published?

Currently, 11 studies based on CCM could be identified and compared. However, the small number shows that CCM is weak in its comprehensive application in the care of the chronically ill [9–19]. At the same time, the number of papers dealing with the systematic treatment of several diseases was rather low. There were not any studies appraising the same patient clientele and similar outcomes. Also, there is the problem that in the literature the terms, CCMs and DMPs were sometimes used synonymously or mixed up. Beyond the period under review, there have been other approaches, such as those of the JA-CHRODIS group with the aim to improve the care for multimorbid chronically ill patients at different levels, based on Wagner's CCM model [30, 31]. However, an overall European approach is still pending. To reflect the European-wide care situation adequately, a uniform definition of terms and a clear declaration of comorbidities will be necessary for the future.

#### 3.2.2. Is There a Uniform Evaluation of the CCM-Based Programs?

**Figure 3:**
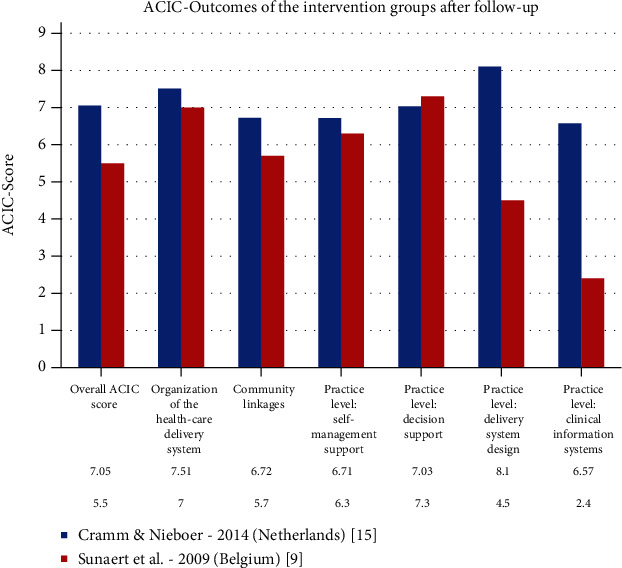
Comparison of the ACIC values.

**Figure 4:**
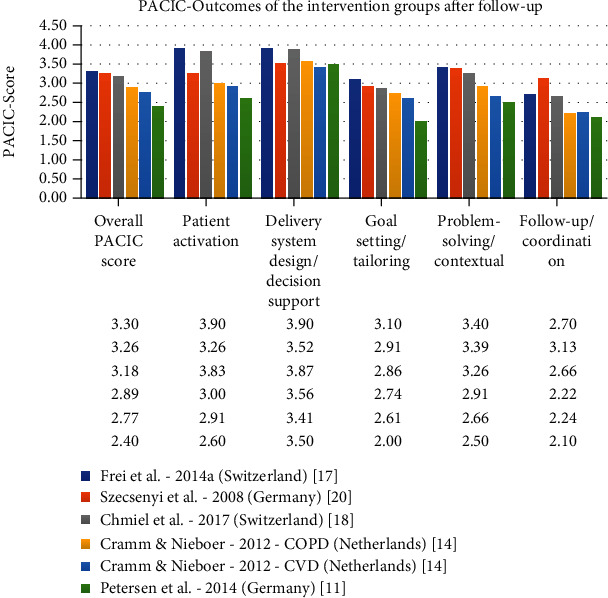
Comparison of the PACIC values.

An evaluation of CCM-based programs is possible insofar that patient and health care worker implementation and perceptions were measured by the “Patient Assessment of Chronic Illness Care” (PACIC) and “Assessment of Chronic Illness Care” (ACIC). The PACIC for the Care of Chronically Ill Patients is a valid self-report instrument to measure the extent to which chronically ill patients receive CCM-based care [32, 33]. The ACIC was established to help organizing groups to pinpoint areas where care for the chronically ill can be improved [32, 34]. It allows assessing the level and type of improvement in the system use rather than measuring traditional outcome measures (e.g., HbA1c values), “process indicators” (e.g., percentage of diabetics receiving eye examinations), or “productivity measures” (e.g., number of treated patients) [15, 32, 33]. Some studies contained neither ACIC nor PACIC results. The trials that used ACIC and PACIC as parameters are shown in Figures 3 and 4, which give an overview of the strengths and weaknesses of the individual programs. Of particular note in the field of ACIC is the study by Cramm and Nieboer [15], which achieved scores in the upper third of the rating scale in all points, reflecting a high level of satisfaction and quality on the part of providers.  Figure 4 clearly shows that the study by Frei et al. [17] stands out from the other interventions in almost all points. The patients perceived the way they were motivated and the maintenance of the care as particularly positive. In addition to the parameters mentioned above, there is an existing approach by Palmer et al. from 2018 [31] that recommended breaking down the existing CCM into an additional 16 components to increase transparency. This would potentially facilitate comparability in the future.

#### 3.2.3. Is Comparability of the Evaluations Possible?

As already described, the field of study proved to be heterogeneous in terms of the types of studies and different settings. Thus, the direct comparison of individual studies with each other is difficult, but the checklist from Table S1 in the supplementary material provides a good overview of the interventions and their settings. In Europe, we found only two randomized control trials by Frei et al. [17] and Muntinga et al. [16], of which the one by Frei et al. [17] stood out in point of PACIC. Most of the studies were longitudinal and cross-sectional. The remaining studies without a control group could not definitively prove the direct relationship between the measured outcomes due to bias and lack of representativeness. Due to the lack of blinding in most of the studies, a distortion of the results by detection bias is possible. As can be seen in Table S1 in the supplementary material, the sizes of the study groups differ significantly both among participants and among providers. Patient numbers ranged from 194 patients in Chmiel et al. [18] to 8574 in Profili et al. [13]. The number of care providers varied from one health center in Musacchio et al. [12] to 483 providers in Profili et al. [13]. Also, the diseases of the patients included varied widely, for example, Cramm and Nieboer [14] focused on COPD and CVD patients, whereas other studies such as Samoutis et al. [19] included only multimorbid diabetics with concomitant diseases. When comparing medical outcomes, the range of studies can be divided into several groups. The first group defined target values based on clinical parameters such as HbA1c, blood pressure (BP), total cholesterol (TC), HDL, and LDL. Other studies, such as by Sunaert et al. [9] and Cramm and Nieboer [15], determined ACIC and did not assume target values. The studies by Petersen et al. [11] and Profili et al. [13] have the collection of patient data beyond ACIC/PACIC in common. The remaining studies by Sunaert et al. [10], Musacchio et al. [12], Frei et al. [17], Chmiel et al. [18], and Samoutis et al. [19] established target values before the study began or were based on specific guidelines. HbA1c as a distinctive parameter was measured in both intervention and control groups in the studies by Sunaert et al. [10], Musacchio et al. [12], Frei et al. [17], Chmiel et al. [18], and Samoutis et al. [19]. In the intervention group, the HbA1c value decreased in all studies except in the studies of Samoutis et al. [19] when the average value remained almost the same (increase in the value for intervention group after a follow-up of 0.01% over time). In the study of Sunaert et al. [10], there was a significant decrease of HbA1c of 0.49% on average, followed by Musacchio et al. [12] with 0.4% in the test group of patients with moderate blood glucose elevation, Chmiel et al. [18] with 0.24%, and Frei et al. [17] with a decrease of 0.2% in the average value. Considering the measured blood BP values, improvement was seen in the study of Frei et al. [17] (intervention: baseline (mmHg) from 140.30 ± 18.40/83.10 ± 10.40 to 136.40 ± 17.50/79.60 ± 9.90 after follow-up). In Chmiel et al. [18], diastolic blood pressure (DBP) for intervention was also lower at follow-up compared with baseline values (T0 mean: 81.65 ± 10.17 mmHg vs. T2 mean: 77.16 ± 9.27 mmHg), whereas systolic blood pressure (SBP) did not change significantly. In Musacchio et al. [12] the change in BP values was less significant, but the rate of patients with high BP values (≥140/90 mmHg) decreased from 62.1% to 58.5% and that of patients with low BP (≤130/85 mmHg) from 24.7% to 23.5%. The study results of Samoutis et al. [19] showed for the intervention group at follow-up a 3.5 mmHg lower mean SBP (*p* = 0.0022) and a 2.3 mmHg lower mean DBP (*p* < 0.0001). In terms of lipid levels, the study of Sunaert et al. [10] improved TC levels significantly (*p* = 0.0021) from 199.07 ± 0.60 mg/dl to 173.94 ± 2.95 mg/dl) in the intervention. Samoutis et al. [19] showed a 0.51 mmol/l lower mean TC (*p* < 0.0001) and a 0.35 mmol/l lower mean LDL (*p* = 0.0022). Still, no statistically significant differences were observed for mean HDL (*p* = 0.11) and mean TG (*p* = 0.82). Musacchio et al. [12] recorded that in the intervention group, the number of patients with high LDL cholesterol (≥130 mg/dl) decreased from 26.6% (24.9%-28.3%) to 19.7% (18.3%-21.1%). The number of patients with low LDL cholesterol (≤100 mg/dl) increased from 39.7% (37.8%-41.6%) to 47.3% (45.5%-49.0%). Frei et al. [17] mentioned that the LDL level of the intervention group decreased from 2.8 ± 1.1 mmol/l to 2.7 ± 1.0 mmol/l. The study of Chmiel et al. [18] described that the LDL target value of 2.6 mmol/l was achieved significantly more often after the follow-up period than at baseline (20% vs. 59%).

## 4. Discussion

### 4.1. Which European CCM-Based Program Was the Most Effective in Terms of Clinical Data, Cost-Effectiveness, and Subjective Perceptions?

The distribution of the studies among the respective countries is very heterogeneous (Table 2). In general, the selected programs almost evenly encompassed all pillars of CCM, with some providing a focus [12, 16] and offering a high degree of scientific quality by referencing guidelines and existing programs [17–19]. In the Netherlands, 7 of the 19 publications were published, which shows that there is an increased interest in functioning CCMs and their improvement [14–16, 21–24]. Switzerland published 5 of the 19 papers in the period in question [17, 25–27]. The RCT study by Frei et al. from 2014 [17] can be considered particularly successful. It achieved high scores in the PACIC score, which the authors based on the fact that patients perceived the changes in care as positive. According to the authors, the program managed an improvement of cardiovascular risk and other clinical data (e.g., BP and LDL) [17]. Due to differences in the various healthcare systems and organizational structure, no model can be transferred as a whole to another setting. In terms of outcome, studies were evaluated either process-oriented or result-oriented or across-the-board. The detailed presentation of the parameters is shown in Table S1 in the supplementary material. However, the regularity of studies of individual parameters was not always guaranteed. The studies by Sunaert et al. [10], Profili et al. [13], and Cramm and Nieboer [15] mentioned an examination of the cost structure in terms of the pay-for-performance method. A detailed look at cost structures and their magnitude was not possible due to insufficient data, as the evaluating studies all focused on the implementation of the care approaches. However, this aspect is of great importance in the future, as cost-efficient and patient-oriented care will be indispensable.

### 4.2. Which Program Can Be Named “Best Practice?” Can a Recommendation Be Made for Europe?

In the context of the present study, it is not possible to identify a model that meets all requirements without exception, primarily because of the inconsistent initial situations in the existing supply systems and the corresponding divergent framework conditions. The existing assessment tools for CCM (ACIC/PACIC) [18] have not been applied to all studies listed and are not sufficiently meaningful for an overall assessment. Concerning the PACIC, which focuses on patients, Switzerland should be highlighted with the studies of Chmiel et al. [18] and Frei et al. [17]. The ACIC, which incorporates the view of the healthcare workers, was only used in the studies of Cramm and Nieboer [15] and Sunaert et al. [9], with the study of Cramm and Nieboer [15] from the Netherlands achieving better values. Compared to the other studies evaluated, the work of Frei et al. [17] showed the highest scores in the areas of “Overall PACIC score,” “Patient activation,” “Delivery system design/decision support,” “Goal setting/tailoring,” and “Problem solving/contextual.” In addition, the study results showed that CCM can be implemented successfully in single-care practices especially in rural regions and improve patient-boosting parameters. Another strength of the study was that it was carried out in a real-life environment and reflects the situation that occurs in most European countries. In general, the study by Frei et al. [17] proved that the CCM approach can be implemented at a reasonable effort and with comparatively little loss of quality in daily primary care for the chronically ill.

### 4.3. Strength and Limitations

#### 4.3.1. Strength

Two renowned databases were used for identification. The studies were assigned subjectively by four independent reviewers according to a clearly defined set of inclusion and exclusion criteria. The desired study design of the RCT was available twice.

#### 4.3.2. Limitations

The majority of the included study designs caused distortions. The comparability of the few selected studies is limited by the heterogeneity of the implementation of the CCM and the different settings. Concrete results could only be identified concerning PACIC and ACIC. The guidelines on medical parameters differ considerably, which does not allow for a uniform assessment. In some studies, absolute values were used; in other situations, the dynamic changes of the parameters were detected. It was difficult to classify the collected data in tables with their adequate specifications. Also, the studies differed greatly in terms of the number of participants and containers, and the issue of financial structuring of the models could only be poorly addressed. The literature search may be incomplete because grey literature was excluded.

## 5. Conclusions

In summary, all the programs listed have made the CCM to the best of their ability. The need for change was recognized, but implementation still has potential and should be critically reviewed. Homogeneous standards for implementing the individual pillars of the CCM would be just as useful as a uniform assessment strategy for the individual subelements. The ACIC and PACIC form a good basis as parameters. Therefore, the checklist of Rothe et al. [8] would, in modified form, be an ideal instrument to examine the other facets of the CCM in detail. The different medical parameters and measured results underline that the CCM is proven to be a universal instrument for patients with complex and various diseases. It is positive that 11 European countries are addressing the disease-independent concept of CCM, but because of demographic change, dissemination and optimization are essential.

## Figures and Tables

**Table 1:** Inclusion and exclusion criteria.

Inclusion criteria	Exclusion criteria
Published between 01/2008 and 05/2018	Lack of quality or ambiguity in operationalization, methods, presentation of results, and analysis of various methods
European primary studies independent of study design	
English language	

**Table 2:** Presentation of the articles according to country and study type.

Belgium	Sunaert et al. [9]	Implementation of a Program for Type 2 Diabetes Based on the Chronic Care Model in a Hospital-Centered Health Care System: “the Belgian Experience”	Longitudinal study
	Sunaert et al. [10]	Effectiveness of the Introduction of Chronic Care Model-Based Program for Type 2 Diabetes in Belgium	Quasiexperimental study

Cyprus	Samoutis et al. [19]	A Pilot Quality Improvement Intervention in Patients with Diabetes and Hypertension in Primary Care Settings of Cyprus	Control study

Germany	Szecsenyi et al. [20]	German Diabetes Disease Management Programs Are Appropriate for Restructuring Care according to the Chronic Care Model: an Evaluation with the Patient Assessment of Chronic Illness Care Instrument	Written interviews
	Petersen et al. [11]	Implementation of Chronic Illness Care in German Primary Care Practices—How Do Multimorbid Older Patients View Routine Care? A Cross-Sectional Study using Multilevel Hierarchical Modeling	Cross-sectional study

Italy	Musacchio et al. [12]	Impact of a Chronic Care Model Based on Patient Empowerment on the Management of Type 2 Diabetes: Effects of the SINERGIA Programme	Longitudinal study
	Profili et al. [13]	Changes in Diabetes Care Introduced by a Chronic Care Model-Based Programme in Tuscany: a 4-Year Cohort Study	Longitudinal study

Netherlands	Ruikes et al. [21]	The CareWell-Primary Care Program: Design of a Cluster-Controlled Trial and Process Evaluation of a Complex Intervention Targeting Community-Dwelling Frail Elderly	Study protocol
	Cramm and Nieboer [14]	The Chronic Care Model: Congruency and Predictors among Patients with Cardiovascular Diseases and Chronic Obstructive Pulmonary Disease in the Netherlands	Cross-sectional study
	Muntinga et al. [24]	Implementing the Chronic Care Model for Frail Older Adults in the Netherlands: Study Protocol of ACT (Frail Older Adults: Care in Transition)	Study protocol
	Spoorenberg et al. [22]	Embrace, a Model for Integrated Elderly Care: Study Protocol of a Randomized Controlled Trial on the Effectiveness regarding Patient Outcomes, Service Use, Costs, and Quality of Care	Study protocol
	Cramm and Nieboer [15]	Short- and Long-Term Improvements in Quality of Chronic Care Delivery Predict Program Sustainability	Longitudinal study
	Spoorenberg et al. [23]	Experiences of Community-Living Older Adults Receiving Integrated Care Based on the Chronic Care Model: a Qualitative Study	Interviews/surveys
	Muntinga et al. [16]	From Concept to Content: Assessing the Implementation Fidelity of Chronic Care Model for Frail, Older people Who Live at Home	RCT

Switzerland	Frei et al. [26]	The Chronic Care for Diabetes Study (CARAT): a Cluster Randomized Controlled Trial	Study protocol
	Zuercher et al. [27]	Baseline Data of a Population-Based Cohort of Patients with Diabetes in Switzerland (CoDiab-VD)	Written interviews
	Frei et al. [17]	Implementation of the Chronic Care Model in Small Medical Practices Improves Cardiovascular Risk but not Glycemic Control	RCT
	Frei et al. [25]	Congruency of Diabetes Care with the Chronic Care Model in Different Swiss Health Care Organisations from the Patients' Perspective: a Cross Sectional Study	Cross-sectional study
	Chmiel et al. [18]	Four-Year Long-Term Follow-Up of Diabetes Patients after Implementation of the Chronic Care Model in Primary Care: a Cross-Sectional Study	Cross-sectional study

## Data Availability

The data supporting this review is from previously reported studies and datasets that have been cited. The processed data are available in the widely used databases or from the corresponding author upon request.

## Supplementary Materials

Supplementary Materials Table S1: checklist to characterize included European CCM programs.
