# Supplementary material for: Chronic-Care-Management Programs for Multimorbid Patients with Diabetes in Europe: A Scoping Review with the Aim to Identify the Best Practice
Source: J Diabetes Res. 2021 Nov 9;2021:6657718. doi: 10.1155/2021/6657718 (PMC8595013; doi:10.1155/2021/6657718)
Supplement: Supplementary Materials — Table S1: checklist to characterize included European CCM programs. [file 6657718.f1.docx]

| Country | Belgium | | Cyprus | Germany | Italy | | Netherlands | | | Switzerland | |
| --- | --- | --- | --- | --- | --- | --- | --- | --- | --- | --- | --- |
| Author | Sunaert  et al.,  2009  [9] | Sunaert  et al.,  2010  [10] | Samoutis  et al.,  2010  [19] | Petersen  et al.,  2014  [11] | Musacchio  et al.,  2011  [12] | Profili  et al., 2017  [13] | Cramm  & Nieboer, 2012  [14] | Cramm  & Nieboer,  2014  [15] | Muntinga  et al.,  2015  [16] | Frei  et al.,  2014  [17] | Chmiel  et al.,  2017  [18] |
| Key  Compo-nents  of the  CCM  program | I to VI | I to VI | I to VI  Focus on  the  introduction  of a new  Clinical  Information  System | I to VI | I to VI  Main focus on:  II  IV  VI | I to VI | I to VI | I to VI | I to VI  geriatric  assessment + multi-disciplinary team  consultation | I to V | I to VI |

I Delivery System Design

II Organization of Healthcare

III Decision Support

IV Self-management Support

V Clinical Information System

VI Community Resources

| Country | Belgium | | Cyprus | Germany | Italy | | Netherlands | | | Switzerland | |
| --- | --- | --- | --- | --- | --- | --- | --- | --- | --- | --- | --- |
| Author | Sunaert  et al.,  2009  [9] | Sunaert  et al.,  2010  [10] | Samoutis  et al.,  2010  [19] | Petersen  et al.,  2014  [11] | Musacchio  et al.,  2011  [12] | Profili  et al., 2017  [13] | Cramm &  Nieboer, 2012  [14] | Cramm  & Nieboer,  2014  [15] | Muntinga  et al.,  2015  [16] | Frei  et al.,  2014  [17] | Chmiel  et al.,  2017  [18] |
| CCM  program? | Yes  CCM  based | Yes  CCM  based | Yes  CCM  based | Yes  based on standard German health care | Yes  SINERGIA-  Programme | Yes  CCM  based | Yes  13 newly  launched CCM- based programs | Yes  CCM  based | Yes  ACT-  Programme  (frail older Adults: Care in Transition) | Yes  CCM  based | Yes  Carat-Programme |
| Has the  program developed button up? | Yes | Yes | No | No | Yes | No | No | No | Yes | Yes | Yes |

| Country | Belgium | | Cyprus | Germany | Italy | | | Netherlands | | | Switzerland | |
| --- | --- | --- | --- | --- | --- | --- | --- | --- | --- | --- | --- | --- |
| Author | Sunaert  et al.,  2009  [9] | Sunaert  et al.,  2010  [10] | Samoutis  et al.,  2010  [19] | Petersen  et al.,  2014  [11] | Musacchio  et al.,  2011  [12] | | Profili  et al., 2017  [13] | Cramm  &  Nieboer, 2012  [14] | Cramm  &  Nieboer,  2014  [15] | Muntinga  et al.,  2015  [16] | Frei  et al.,  2014  [17] | Chmiel  et al.,  2017  [18] |
| Participa-tion rate  of  physicians? | 49% of GPs  Approx. 20% of the  diabetes popular-tion | GP to  popula-tion ratio:  Intervention:  1:972  Control: 1:1069 | --- | --- | --- | 483 out of 2700 GPs (17.89%)  After 2010, further GPs joined them (60% in 2014) | | --- | T0 (2010): 218  (55.00% response rate)  T2 (2012): 265  (63.00% response rate) | --- | Around 800 randomly selected PCPs were invited to an informa-tion meeting on the study  [17]  Interven-tion: 30 PCPs | Start of studies:  30 PCPs  326  patients  Follow-up:  24 PCPs  (80.00%)  194  patients (64.00%) |
| Guidelines for patients are the  scientific  basis | Yes | Yes | Yes | Yes | Yes | Yes | | Yes | Yes | Yes | Yes | Yes |

| Country | Belgium | | Cyprus | Germany | Italy | Netherlands | | | | Switzerland | |
| --- | --- | --- | --- | --- | --- | --- | --- | --- | --- | --- | --- |
| Author | Sunaert  et al.,  2009  [9] | Sunaert  et al.,  2010  [10] | Samoutis  et al.,  2010  [19] | Petersen  et al.,  2014  [11] | Musacchio  et al.,  2011  [12] | Profili  et al., 2017  [13] | Cramm  &  Nieboer, 2012  [14] | Cramm  & Nieboer,  2014 [15] | Muntinga  et al.,  2015  [16] | Frei  et al.,  2014  [17] | Chmiel  et al.,  2017  [18] |
| Complex guidelines for patients with DMT2 and multiple conditions are available? | Not  exactly  defined | Not  exactly  defined | Yes  …HTN management guidelines, ADA,  St. Vincent Declaration guidelines,  lipid guidelines based on the National Cholesterol Education Program III…[19] | Not  exactly  defined | Not  exactly  defined  … multi-faceted framework for  enhancing healthcare delivery… | Not  exactly  defined  … multi-professio-nal interventions… | Not  exactly  defined | Not  exactly  defined | Not  exactly  defined | Yes  men-tioned in study protocol | Yes  men-tioned in study protocol |
| Integrated care  structure | Yes | Yes | Practice  teams  only | Yes | Yes | Yes | Yes | Yes | Yes | Yes | Practice teams only |

| Country | Belgium | | Cyprus | Germany | Italy | Netherlands | | | | Switzerland | |
| --- | --- | --- | --- | --- | --- | --- | --- | --- | --- | --- | --- |
| Author | Sunaert  et al.,  2009  [9] | Sunaert  et al.,  2010  [10] | Samoutis  et al.,  2010  [19] | Petersen  et al.,  2014  [11] | Musacchio  et al.,  2011  [12] | Profili  et al.,  2017 [13] | Cramm  &  Nieboer, 2012 [14] | Cramm  &  Nieboer,  2014 [15] | Muntinga  et al.,  2015  [16] | Frei  et al.,  2014  [17] | Chmiel  et al.,  2017  [18] |
| Outcome  orientated  quality  management | Yes  evalu-ation based on the  ACIC | Yes  process- and result- oriented  (e.g., ACIC) | Yes  process- and result- oriented | Yes  evaluation based on the  PACIC | Yes  result-  oriented | Yes  process- and result-oriented | Yes  evalu-ation based on the PACIC | No  process- orientated | Yes  process- and result-oriented | Yes  evalu-ation based on the  PACIC | Yes  evalu-ation based on the  PACIC |
| Patient  centered  approach | No | Yes | Yes | Yes | Yes | Yes | Yes | No | Yes | Yes | Yes |
| Regulary blood  glucose self-  management | No | No | Yes | Yes | Yes | Yes | No | No | No | Yes | Yes |

| Country | Belgium | | Cyprus | Germany | Italy | Netherlands | | | | Switzerland | |
| --- | --- | --- | --- | --- | --- | --- | --- | --- | --- | --- | --- |
| Author | Sunaert  et al.,  2009  [9] | Sunaert  et al.,  2010  [10] | Samoutis  et al.,  2010  [19] | Petersen  et al.,  2014  [11] | Musacchio  et al.,  2011  [12] | Profili  et al.,  2017  [13] | Cramm  &  Nieboer, 2012 [14] | Cramm  &  Nieboer,  2014 [15] | Muntinga  et al.,  2015  [16] | Frei  et al.,  2014  [17] | Chmiel  et al.,  2017  [18] |
| Quatery  Examina-tions  (e.g. HbA1c,  body-weight) | No | No | No | No | six-monthly | No | No | No | No | measure-ments every 4 months | No |
| Annual  Examin-ations | No | No | Yes  -BP  -TC  -HDL  -LDL  -urine-  protein  testing  -dilated eye and foot examination | No | Yes | Yes  -BP  -waist-  circum-  ference  -Hb1Ac  -TC  -electro-lytes  -protein-  uria  -HbA1c  -BMI | No | No | Yes | Yes  e.g.  -HbA1c  -SBP  -DBP  -LDL  -BMI  -HDL  -TC  -waist/hip  ratio  -PACIC | Yes  e.g.  -HbA1c  -LDL  -exa-minations  e.g.  food-control,  neuro-logical tests and  eye exa-minations |

| Country | | Belgium | | | Cyprus | | | Germany | | Italy | | | | Netherlands | | | | | Switzerland | | | |
| --- | --- | --- | --- | --- | --- | --- | --- | --- | --- | --- | --- | --- | --- | --- | --- | --- | --- | --- | --- | --- | --- | --- |
| Author | | Sunaert  et al.,  2009  [9] | | Sunaert  et al.,  2010  [10] | Samoutis  et al.,  2010  [19] | | | Petersen  et al.,  2014  [11] | | Musacchio  et al.,  2011  [12] | | Profili  et al.,  2017  [13] | | Cramm  & Nieboer, 2012 [14] | Cramm  & Nieboer,  2014 [15] | | | Muntinga  et al.,  2015  [16] | Frei  et al.,  2014  [17] | | Chmiel  et al.,  2017  [18] | |
| Regular  Education (HbA1c, body weight….) | | No | | Yes | Yes | | | No | | Yes | | Yes | | No | Yes | | | No  Staff  education  only | No  Staff education only | | No  Staff education only | |
| Hospitalization  rate | | No | | Yes | No | | | No | | No | | Yes  reduction was only registered in urgent cases of long-term neurolo-gical compli-cations | | No | No | | | No | No | | No | |
| Cost effectiveness of the  management  program:  Pay-for-Performance?  Pay for outcome? | | No | | Yes  Pay-for-Perfor-mance | No | | | No | | No | | Yes  Pay-for-  Perfor-mance | | No | Yes  Pay-for-Perfor-mance | | | No | No | | No | |
| Country | Belgium | | | | | Cyprus | Germany | | Italy | | | | Netherlands | | | | | | Switzerland | | |  |
| Author | Sunaert  et al.,  2009  [9] | | Sunaert  et al.,  2010  [10] | | | Samoutis  et al.,  2010  [19] | Petersen  et al.,  2014  [11] | | Musacchio  et al.,  2011  [12] | | Profili  et al.,  2017  [13] | | Cramm  &  Nieboer, 2012  [14] | | | Cramm  &  Nieboer,  2014  [15] | Muntinga  et al.,  2015  [16] | | Frei  et al.,  2014  [17] | Chmiel  et al.,  2017  [18] | |  |
| The diabetes management program involves:  general practitioner, diabetes specialist in own practice,  diabetes specialist in hospital, nurses,  specialists for diabetic complication etc. | -GPs  -medical  specialists  -pharma-cists  -nutrition-ists  -podi-atrists  -nurses  -home care | | -GPs  -special-ists  -phar-macists  -nutri-tionists  -podo-logists  -nurses | | | -PCPs  -nurses  -patients from the interven-tion  centres | -GPs  -nurses | | Diabetes team:  -hospitals,  -diabeto-logists  -nurses  -tele-medical resources | | -GPs  -nurses  -health workers | | -GPs  -physio-  therapists | | | -GPs  -doctors  -nurses  -nutritio-nists  -physio-therapists  -etc. | -GPs  -practice -nurses  -geriatric  expert  teams  -phar-macists  -nursing  staff | | -PCPs  -nurses  -exter-nal  labor-atories | -GPs  -physio-therapists  -dietici-ans | |  |

| Country | Belgium | | Cyprus | Germany | Italy | | Netherlands | | | Switzerland | |
| --- | --- | --- | --- | --- | --- | --- | --- | --- | --- | --- | --- |
| Author | Sunaert  et al.,  2009[9] | Sunaert  et al.,  2010[10] | Samoutis  et al.,  2010 [19] | Petersen  et al.,  2014[11] | Musacchio  et al.,  2011[12] | Profili  et al.,  2017[13] | Cramm  & Nieboer, 2012 [14] | Cramm  &  Nieboer,  2014 [15] | Muntinga  et al.,  2015  [16] | Frei  et al.,  2014  [17] | Chmiel  et al.,  2017  [18] |
| Target  values | --- | - HbA1c ≤ 7.50%  - TG ≤ 2.15  mmol/l | --- | --- | - HbA1c ≤ 7.00%  - BP ≤ 130⁄85 mmHg  - LDL-C  < 2.60 mmol/l | --- | --- | --- | --- | reduction of HbA1c by 0.5% | HbA1c ≤ 7.00%  BP <140/90 mmHg  LDL-C: 2.60 mmol/l |
| Criteria for interme-diate result Quality:  -HbA1c - Baseline | --- | Baseline 2003:  Interven-tion:  7.55%  Control: 7.44% | Baseline:  Interven-tion: 6.82%  Control:  6.88% | --- | Target value for patients in groups- Baseline:  -under ≤ 7.50% (83.40% of participants): 6.60 %  -7.50 to 8.00: 7.70%  -8.00 to 9.00: 8.40 %  -over 9.00: 10.00% | --- | --- | --- | --- | Baseline (01.2010-04.2010:  Interven-tion: 7.8%  Control: 7.6% | Baseline (01.2010-04.2010):  Interven-tion:  7.74%  Control: 7.74% |

| Country | Belgium | | Cyprus | Germany | Italy | | Netherlands | | | Switzerland | |
| --- | --- | --- | --- | --- | --- | --- | --- | --- | --- | --- | --- |
| Author | Sunaert  et al.,  2009[9] | Sunaert  et al.,  2010[10] | Samoutis  et al.,  2010 [19] | Petersen  et al.,  2014[11] | Musacchio  et al.,  2011[12] | Profili  et al.,  2017[13] | Cramm& Nieboer, 2012  [14] | Cramm  &  Nieboer,  2014 [15] | Muntinga  et al.,  2015  [16] | Frei  et al.,  2014  [17] | Chmiel  et al.,  2017  [18] |
| Criteria for Inter-mediate result Quality:  HbA1c - Follow-up | --- | Follow-up 2007:  Interven-tion:  7.06%  Control: 6.90% | Follow-up after 18 months:  Interven-tion: 6.83%.  Control: 7.03 % | --- | Target value for patients in groups –  Follow up:  - under ≤ 7.50% (83.40% of participants):  6.80%  - 7.50% to 8.00%: 7.30%  - 8.00% to 9.00%: 7.40%  - over 9.00%: 7.80% | --- | --- | --- | --- | Follow-up (04.2010-05.2011):  Interven-tion:  7.6%  Control: 7.3% | Follow-  up (01.2011-04.2011):  Interven-tion: 7.51%  Control: 7.65% |

| Country | Belgium | | Cyprus | | Germany | Italy | | Netherlands | | | Switzerland | |
| --- | --- | --- | --- | --- | --- | --- | --- | --- | --- | --- | --- | --- |
| Author | Sunaert  et al.,  2009 [9] | Sunaert  et al.,  2010 [10] | Samoutis  et al.,  2010 [19] | Petersen  et al.,  2014 [11] | | Musacchio  et al.,  2011 [12] | Profili  et al.,  2017 [13] | Cramm & Nieboer, 2012 [14] | Cramm & Nieboer,  2014 [15] | Muntinga  et al.,  2015[16] | Frei  et al.,  2014 [17] | Chmiel  et al.,  2017 [18] |
| Criteria for intermediate Outcome  -BP (< 140/85 mmHg) | --- | --- | Baseline:  SBP  Intervention: 134.14  Control:  135.93  DBP  Intervention: 78.47  Control: 80.00  Follow-up:  SBP  Intervention: 133.56  Control: 137.10  DBP  Intervention: 77.90  Control: 80.29 | | --- | BP ≤ 130/85:  Baseline: 24.70%  Follow-up: 23.50% | --- | --- | --- | --- | Baseline: SBP  Intervention: 140.3  Control: 137.8  DBP  Intervention: 83.1  Control:  78.7  Follow-up:  SBP  Intervention: 136.4  Control: 137.5  DBP  Intervention: 79.6  Control:  79.2 | Baseline: SBP  Intervention: 140.34  Control: 140.34  DBP  Intervention: 81.65  Control: 81.65  Follow-up: SBP  Intervention: 138.36  Control: 135.54  DBP  Intervention: 78.51  Control:  75.91 |

| Country | Belgium | | Cyprus | Germany | Italy | | Netherlands | | | Switzerland | |
| --- | --- | --- | --- | --- | --- | --- | --- | --- | --- | --- | --- |
| Author | Sunaert  et al.,  2009 [9] | Sunaert  et al.,  2010 [10] | Samoutis  et al.,  2010 [19] | Petersen  et al.,  2014 [11] | Musacchio  et al.,  2011 [12] | Profili  et al.,  2017 [13] | Cramm &  Nieboer, 2012 [14] | Cramm & Nieboer,  2014 [15] | Muntinga  et al.,  2015[16] | Frei  et al.,  2014 [17] | Chmiel  et al.,  2017 [18] |
| Criteria for intermediate Outcome  -TC (mmol/l) | --- | Baseline:  Intervention: 199.07  Control: 199.44  Follow-up:  Intervention: 173.00  Control: 180.60 | Baseline:  Intervention: 2.25  Control:  2.09  Follow-up:  Intervention: 2.16  Control:  2.20 | --- | --- | --- | --- | --- | --- | --- | ---- |

| Country | Belgium | | Cyprus | Germany | Italy | | Netherlands | | | Switzerland | |
| --- | --- | --- | --- | --- | --- | --- | --- | --- | --- | --- | --- |
| Author | Sunaert  et al.,  2009 [9] | Sunaert  et al.,  2010 [10] | Samoutis  et al.,  2010 [19] | Petersen  et al.,  2014 [11] | Musacchio  et al.,  2011 [12] | Profili  et al.,  2017 [13] | Cramm &  Nieboer, 2012 [14] | Cramm & Nieboer,  2014 [15] | Muntinga  et al.,  2015[16] | Frei  et al.,  2014 [17] | Chmiel  et al.,  2017 [18] |
| Criteria for intermediate Outcome  -HDL-C (males > 1.1 mmol/l (43 mg/d) females > 1.3 mmol/l (50mg/dl)) | --- | --- | Baseline:  Intervention: 1.24  Control: 1.31  Follow-up:  Intervention: 1.22  Control: 1.30 | --- | --- | --- | --- | --- | --- | --- | --- |
| Criteria for intermediate Outcome  -LDL-C < 2.6 mmol/l (<100mg/dl) | --- | --- | Baseline:  Intervention: 3.45  Control: 3.50  Follow-up  Intervention: 3.28  Control: 3.64 | --- | Baseline: 39.7%  Follow-up: 2  3.5% | --- | --- | --- | --- | Baseline:  Intervention: 2.8  Control:  2.5  Follow-up:  Intervention: 2.7  Control:  2.6 | Baseline:  Intervention: 2.58  Control: 2.58  Follow-up:  Intervention: 2.69  Control: 2.37 |

| Country | Belgium | | Cyprus | Germany | Italy | | Netherlands | | | Switzerland | |
| --- | --- | --- | --- | --- | --- | --- | --- | --- | --- | --- | --- |
| Author | Sunaert  et al.,  2009  [9] | Sunaert  et al.,  2010  [10] | Samoutis  et al.,  2010  [19] | Petersen  et al.,  2014 [11] | Musacchio  et al.,  2011 [12] | Profili  et al.,  2017 [13] | Cramm  &  Nieboer, 2012  [14] | Cramm & Nieboer,  2014  [15] | Muntinga  et al.,  2015  [16] | Frei  et al.,  2014  [17] | Chmiel  et al.,  2017  [18] |
| Criteria for long term outcome quality  -Major limb  amputations  reducing  -Myocardial  infarct rates  reducing  -Stroke rates  reducing  -Cardiovascular  mortality rates reducing  -Microangiopathy rates  reducing  -etc. | --- | --- | -microalbumin/ 24h  -urine protein testing  -dilated eye and foot  examinations  …the annual percentage […] increased from 0% to 37% (n = 103), 59% (n = 164) and 73% (n = 202), respectively.  The comparisons were adjusted for age, gender, baseline values…[19] | --- | --- | -cardio-vascular-complications  -neurological complications  -ophthalmic complications  -renal  diseases  -endocrine/  metabolic  effects  -amputations of lower  extremities  -acute  cardio-  cerebro-  vascular complications | --- | --- | ---- | cardio-  vascular  risk  profile | --- |
